# Topic Specificity and Antecedents for Preservice Biology Teachers’ Anticipated Enjoyment for Teaching About Socioscientific Issues: Investigating Universal Values and Psychological Distance

**DOI:** 10.3389/fpsyg.2020.01536

**Published:** 2020-07-24

**Authors:** Alexander Georg Büssing, Jacqueline Dupont, Susanne Menzel

**Affiliations:** ^1^Working Group of Biology Education, Institute of Science Education, Leibniz University Hannover, Hanover, Germany; ^2^Didactics of Biology, Department of Biology/Chemistry, Osnabrück University, Osnabrück, Germany

**Keywords:** teaching emotion, appraisal, enjoyment, values, psychological distance, teacher identity, teacher professional competence

## Abstract

Enjoyment for teaching represents one of the most frequently reported teaching emotions and positively affects student outcomes. Therefore, researchers and teacher educators need to understand its nature and underlying appraisal processes to prepare motivated teachers as part of initial teacher education. Using cross-sectional questionnaire data from 189 German biology preservice teachers (73.5% female, mean_age_ = 23.45 years, *SD*_age_ = 3.71 years), we empirically tested the topic-specific structure and antecedents of participants’ anticipated enjoyment for teaching. We adapted the established Teacher Emotion Scale to measure preservice teachers’ trait-based enjoyment for teaching by reframing the items with the environmental socioscientific issues of the return of wild wolves and climate change and the health socioscientific issue of preimplantation genetic diagnosis. Confirmatory factor analysis confirmed the best fit of a topic-specific model. We also found different correlations for the anticipated enjoyment for teaching about the issues, but no significant differences in means. Concerning further topic-specific antecedents, the environmentally oriented basic value of universalism predicted the anticipated enjoyment for teaching about the return of wolves, and the socially oriented universal value of benevolence predicted the anticipated enjoyment for teaching about preimplantation genetic diagnosis. Both values inconsistently predicted the anticipated enjoyment for teaching about climate change. While this is in line with the complex nature of this socioscientific issue, psychological distance was a predictor for the anticipated enjoyment for teaching about every topic. While these effects remained stable when controlling for demographic variables, male participants showed a higher anticipated enjoyment for teaching about wolves and about climate change, and female preservice teachers for teaching about preimplantation genetic diagnosis. Further studies are needed to investigate if the results can be transferred to in-service teachers or to other teaching emotions. Furthermore, future studies could examine effects on other factors relevant to teaching emotions such as reactions to student behavior, which have been described as central for the causation of teaching emotions in prior studies (i.e., “reciprocal model of teaching emotions”). The present study stimulates such new studies and adds important knowledge to the understanding of topic specificity and topic-specific antecedents of anticipated enjoyment for teaching, which are relevant for teacher education and professional development.

## Introduction

The preparation of motivated teachers is one of the central goals of initial teacher education, as teaching motivation has been found to be one criterion for teacher competence (Kunter et al., 2013). Because of this, many studies have investigated how teaching motivation can be conceptualized and the way it emerges. Within the framework of professional action competence, teaching motivation was described as “enthusiasm for teaching” (Kunter et al., 2013). This enthusiasm has been found to be linked to student enjoyment through the enthusiasm students perceive when their teachers teach (Frenzel et al., 2009a; Keller et al., 2014). This increased student enjoyment in turn positively affects students’ learning and achievement (Trigwell et al., 2012; Frenzel, 2014). In other studies, teacher enthusiasm also was linked to student performance (Mahler et al., 2018).

Therefore, teacher education has the central goal of preparing teachers to be able to enthusiastically teach about their subject. As prior studies have shown that enjoyment represents the needed internal affective state encompassing enthusiastic teaching (Keller et al., 2016), and anticipated enjoyment is positively connected to teaching motivation (Büssing et al., 2019d), there is a need for further knowledge about the nature of and antecedents of enjoyment for teaching. This especially concerns preservice teachers’ emotions, as the anticipated emotions for teaching may affect the emotions others perceive in real situations (Sutton and Wheatley, 2003).

In prior studies, the emergence of teaching enjoyment often was explained by teachers’ evaluations of student behavior, such as student motivation and discipline (Frenzel, 2014; Becker et al., 2015; Frenzel et al., 2020). In combination with explicit and implicit goals for student behavior, teachers evaluate situations based on specific appraisal dimensions such as goal consistency, coping potential, and goal importance (Frenzel, 2014). This overall approach led to the “reciprocal model of causes and effects of teacher emotions” (Frenzel, 2014, p. 506), with students’ behavior influencing teaching emotions, as well as their instructional behaviors, which are *vice versa* shaping students’ behavior (Frenzel, 2014). While this approach seems reasonable for in-class state emotions, there may be other factors that affect how teachers appraise specific situations.

In particular, there may be further personality-related variables that may affect whether or not a specific teaching situation is appraised to be beneficial (Montag and Panksepp, 2017). This especially concerns topics that are emotionally loaded, such as controversial issues from the environmental or health domains. For example, teachers will evaluate the situation of teaching about climate change as very negative if they possess a contradicting underlying belief system, caused by values or negative attitudes toward the issue (Plutzer et al., 2016). Besides these beliefs, the perceived distance from specific topics may affect teaching emotions; for example, a topic that is perceived as very close to was found to elicit stronger emotions than a topic that is perceived as far away (Van Boven et al., 2010).

While prior studies investigated how closely values may be connected to the experience of specific emotions in everyday life (Nelissen et al., 2007; Tamir et al., 2016), there have been only few investigations of connections between personal values or psychological distance with teaching emotions. From the perspective of appraisal theory, such investigations would be interesting, as values that, for example, correspond to a life goal to protect the environment will be more relevant for teaching in topics that relate to environmental degradation such as biodiversity reduction (Büssing et al., 2019c).

To investigate these dimensions, teaching enjoyment needs to be investigated from a more topic-specific perspective, as it is not possible to investigate topical differences with measures that are not sensible for such differences. While the further topic specificity of teaching emotions has often be debated (Frenzel et al., 2016), a more topic-specific approach to emotions could probably also be able to inform the reciprocal model of teaching emotions. For example, with a more topic-specific approach to emotions, it would be possible to explain differences in teachers’ appraisal of the same student misbehavior (stimulus) in a lesson with a topic positively evaluated by the teacher (situation A) in comparison to a negatively valued topic (situation B).

While several studies showed how personality-related variables such as values, beliefs, and closeness to issues may predict the anticipated enjoyment for teaching (Büssing et al., 2019b, c), there have been no comparative studies yet in which the same participants have been assessed for their anticipated enjoyment for teaching about several topics.

To close these gaps, the present study investigates (1) if preservice teachers’ anticipated enjoyment can be assessed with a topic-specific approach comparing three contrasting topics and (2) if selected universal values and psychological distance serve as antecedents of this topic-specific anticipated enjoyment for teaching.

To underpin our study, we first explain how appraisal theories are helpful for understanding the elicitation of teaching emotions and then define enjoyment for teaching, review prior approaches of explaining the topic specificity of enjoyment and the causation of this emotion, and propose testable hypotheses for universal values and psychological distance as antecedents of anticipated enjoyment for teaching. Finally, we describe the selected issues in detail.

### Appraisal Theory as the Basis for Explaining the Causation of Teaching Emotions

As stated above, within teaching emotion research, appraisal theories have become the main theoretical approach for describing the elicitation of teaching emotions (Frenzel, 2014; Keller et al., 2014). Appraisal theorists define an emotion as a process in which situational stimuli are evaluated for the well-being of the individual (Moors et al., 2013). This evaluation often happens unconsciously (Moors, 2010). For the appraisal that is most relevant for teaching emotions, Frenzel (2014) describes the appraisal of student behavior as a central tenet for the elicitation of teaching emotions.

**FIGURE 1:**
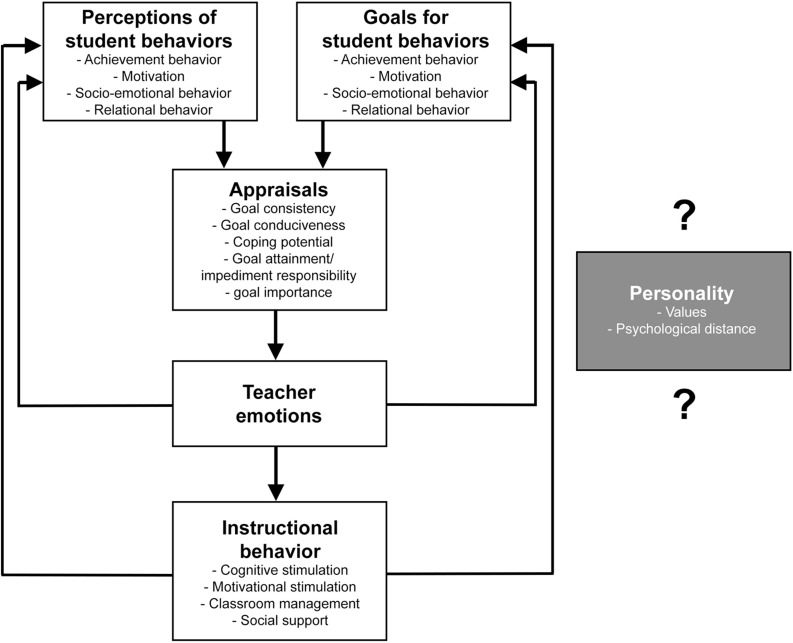
Overview of the reciprocal model on causes and effects of teacher emotions (adapted from Frenzel, 2014) in white rectangles and personality as a missing concept in the gray rectangle.

Generally, the appraisal of student behavior is grounded in teachers’ perceptions of student behavior such as their motivation or relational behavior in combination with teachers’ goals for the respective student behaviors (Becker et al., 2015). In combination of these perceptions and goals, teachers appraise specific teaching situations based on five central appraisal dimensions. Among these, teachers appraise teaching situations based on students’ *goal consistency*, which describes how consistent students behaviors are with a teacher’s goals (Frenzel, 2014). Similarly, *goal conduciveness* refers to the teachers’ appraisal if the student behavior contributes to the achievement of specific classroom goals, such as support of students to reach lesson goals (Becker et al., 2015). Based on these two and three other appraisal dimensions, teachers experience specific teaching emotions. These teaching emotions not only affect teachers’ perceptions and goals for student behavior, but also teachers’ instructional behavior such as cognitive or motivational stimulation (Frenzel, 2014). As this instructional behavior affects the perception and goals for students behavior, the model was called the “reciprocal model of teaching emotion” (Frenzel, 2014, p. 506; Frenzel et al., 2018, 2020). The model is presented in Figure 1.

As described in Figure 1, it is yet unclear if personality-related goals and beliefs, such as universal values or psychological distance, may affect the occurrence of teaching emotions. From the perspective of appraisal theory, a connection to more abstract values seems reasonable, as prior studies have shown how general values are connected to the experience of discrete emotions (Nelissen et al., 2007; Tamir et al., 2016). Furthermore, Schutz (2014) has already described beliefs, which are based on specific value systems (Whittaker et al., 2006), as important to consider for teaching emotions (Schutz, 2014). But at the moment, studies that explicitly investigate connections between specific sets of universal values and teaching emotions are missing. To test this assumption, we explicitly selected one specific emotion, for which there is already a large research basis on its underlying appraisal processes available.

### Anticipated Enjoyment for Teaching—Definition, Antecedents, and Topic Specificity

Generally, *enjoyment for teaching* represents the internal state of subjective happiness and approach motivational tendencies towards teaching (Frenzel et al., 2016). As enjoyment constitutes an internal foundation for enthusiastic teaching (Frenzel et al., 2009a), there is a need for further knowledge about what contributes to teachers’ experience of enjoyment for teaching.

Following the control-value theory of achievement emotions, emotions may be concentrated on prospective or retrospective outcomes, or on the activity itself (Pekrun, 2006). For teacher education, preservice teachers may already imagine themselves in classrooms and associate specific emotions with this situation. In this study, we refer to *anticipated enjoyment for teaching*, if someone envisions themselves teaching in the future and enjoying this activity.

Even though prior studies showed that enjoyment for teaching may not differ empirically between preservice and in-service teachers (Lohbeck et al., 2018), there may be severe differences between preservice and in-service teachers’ *anticipated* enjoyment for teaching. However, the investigation of preservice teachers’ prospective emotions could have important conclusions for teacher education, which is why this study concentrates on anticipated enjoyment toward teaching.

As mentioned above, prior studies primarily investigated student behavior as a cause of enjoyment for teaching (Frenzel, 2014). For example, a quantitative study found students’ ratings of class motivation as predictors of teacher enjoyment, mediated by teacher appraisals (Becker et al., 2015). This fits older results, which showed how ratings of perceived student behaviors such as performance, motivation, and discipline predicted teachers trait and state enjoyment for teaching (Frenzel et al., 2009b).

Besides these external variables, other studies showed how also internal variables such as efficacy beliefs were connected to enjoyment for teaching (Hagenauer et al., 2015). Several studies replicated these results or found similar connections between efficacy beliefs and emotions. For example, one study illustrated how the perceived self-efficacy of preservice teachers in a teaching practicum was a predictor of enjoyment for teaching (Hascher and Hagenauer, 2016). A connection between these variables was also found in more specialized topics such as science education (Brígido et al., 2013). But while all these studies investigated how either classroom conditions or specific efficacy beliefs may affect enjoyment for teaching, there is only scarce research about the topic-specific dimensions of these teaching events, even if the results of other studies may have implied this.

For example, in one study, in-service teachers filled out emotion diaries and reported on their experienced emotions of enjoyment, anger, and anxiety (Frenzel et al., 2015). When considering the variance that was explained at the teacher, student group, and class-period level, most of the variance (approximately 62%) of enjoyment was explained at the class-period level (Frenzel et al., 2015). This means that a lot of variation is explained by more situational factors that differ between class periods. Besides the behavior of students within individual classes, this variance also includes several topics that may differ between class periods. As the study measured emotions not on a topic-specific level, it is not possible to disentangle the variance that is explained by the topics on this level. To explicitly investigate this, we propose our first hypothesis.

**Hypothesis 1 (H_1_):** Anticipated enjoyment for teaching can be meaningfully measured in a topic-specific manner.

### Additional Antecedents of Topic-Specific Anticipated Enjoyment for Teaching

#### Universal Values of Universalism and Benevolence

As described above, appraisal processes rely on often implicit cognitive evaluations of specific situations. These evaluations are affected by prior experiences, worldviews, values, and personal goals (Pekrun, 2006; Schutz et al., 2006). This has been acknowledged in prior research about teaching emotions, with the exception of those only taking into account goals for student behavior (e.g., Chang, 2013; Frenzel, 2014; Becker et al., 2015), but these typically neglect more abstract goals, such as the prosperity of the earth’s environment or the well-being of other people. These goals may be especially relevant within the teaching of Education for Sustainable Development (ESD), which describes an educational program of the United Nations aiming for sustainable development of planet Earth in concordance of ecological, economic, and social dimensions (Leicht et al., 2018).

Empirically, such values have been investigated in the light of the theory of universal human values from Schwartz (1994). In this theory, every human’s underlying values may be described by 10 different value dimensions, which may be combined into higher-order clusters (Schwartz, 1994). Within environmental topics, several studies already showed the relevance of universal values, for example, from the cluster of self-transcendence (Menzel and Bögeholz, 2010). This cluster includes the well-being of the Earth, as well as other people, and is further distinguished into the values of universalism and benevolence (Schwartz, 1994). While *universalism* depicts the value of “understanding, appreciation, tolerance, and protection of nature for the welfare of all people and for nature” (Schwartz, 1994, p. 22), *benevolence* concentrates on the “preservation and enhancement of the welfare of all people with whom one is in frequent personal contact” (Schwartz, 1994, p. 22).

Several studies started the investigation of the connections between values and emotions for explaining human behavior (Brosch and Sander, 2014) and found connections between basic human values and the reported general frequency of emotions in daily life (Nelissen et al., 2007). Furthermore, universalism and benevolence also predicted emotions and other prosocial tendencies such as empathic concern and perspective taking in a quantitative study (Silfver et al., 2008). Prior studies also showed how universalism was connected to proenvironmental motivations (Schultz, 2005; Gifford and Nilsson, 2014). These results dovetail nicely onto research about teacher emotions.

One study explicitly investigated connections between universalism and anticipated enjoyment for teaching in the domain of teaching inclusion (Büssing et al., 2019b). In this study, preservice teachers with a more universalistic value orientation showed higher anticipated enjoyment for teaching in inclusive settings. Similarly, a study found more specialized wildlife value orientations as predictors of anticipated enjoyment for teaching about wild wolves (Büssing et al., 2019c). Other studies showed how emotions and teacher identity may be strongly connected with each other (O’Connor, 2008). For example, a qualitative study illustrated how positive emotions based on positive experiences build the foundations of professional identity and contribute to professional development (Timoštšuk and Ugaste, 2012).

As values may be used as empirical indicators of identity (Hitlin, 2003), the selected universal values paradigmatically capture teachers’ identities for two contextual domains of preserving the welfare of nature (universalism) and other people (benevolence). Preservice teachers with a general universalistic worldview may, sometimes unconsciously, also transfer this worldview into their teaching, which is why we hypothesize the values of universalism and benevolence to be connected to the appraisal of anticipated enjoyment for teaching. Given the topical domains, universalism will be a predictor for environmental topics and benevolence in people-related topics from domains such as health.

**Hypothesis 2 (H_2_):** Universalism is a positive predictor for anticipated enjoyment for teaching about ecological topics.

**Hypothesis 3 (H_3_):** Benevolence is a positive predictor for anticipated enjoyment for teaching about health topics.

#### Psychological Distance

Besides values as a personality-related variable, prior studies illustrated the connection between psychological distance and emotions (Van Boven et al., 2010). *Psychological distance* refers to the perceived distance to specific objects, events, or actions (Liberman and Trope, 2008; McDonald et al., 2015) and is constituted by the four dimensions of temporal, spatial, social, and hypothetical distance (Liberman and Trope, 2014). This means that people will feel close to an object or process if this process concerns them personally, within their close spatial surrounding, in an immediate moment, and its occurrence is assessed as very likely. This closeness may be connected to feelings of relevance, as more close objects may also be evaluated as more relevant (Liberman and Trope, 2014).

Personal relevance within a respective situation was found to be a general prerequisite for an emotional reaction, as only situations evaluated as relevant will lead to an emotional reaction at all (Scherer, 2005). Furthermore, prior studies showed that personal relevance increases emotional reactions (Harmon-Jones et al., 2006) and may be connected to intrapersonal variables such as reasoning (Caparos and Blanchette, 2017). However, only a few studies have explicitly investigated connections between psychological distance and teaching motivation.

For example, one study found psychological distance as a predictor of anticipated enjoyment for teaching in the topic of the return of wild wolves (Büssing et al., 2019c). Similarly to this, another study investigated how personal experiences of cancer may affect in-service teachers’ motivation to teach about the disease (Heuckmann et al., 2020). Therefore, we hypothesize that the psychological distance to specific topics is connected to the anticipated enjoyment for teaching about the respective topic. As a smaller distance may induce a higher closeness, we hypothesize a negative relationship.

**Hypothesis 4 (H_4_):** Psychological distance is a negative predictor for anticipated enjoyment for teaching.

### Selection of Suitable Socioscientific Teaching Topics

As described before, a more fine-grained approach to teaching enjoyment requires specific topics. Only few studies have explicitly addressed such a topic-specific approach to emotions. Besides the already mentioned studies, for example, about returning wolves, one other study selected climate change as a topic and found connections between emotions toward the general topic, perceptions of plausibility, and emotions toward teaching (Lombardi and Sinatra, 2013).

In science education, topics that include a strong social debate such as climate change can be described as *socioscientific issues*, which generally represents a progressive teaching approach facilitating student discussion and decision-making focused on controversial scientific issues (Zeidler, 2014). Such controversial issues describe open-ended learning problems recurring to deeply rooted conflicts between a substantial number of people (Levinson, 2006). As these conflicts may finally be not solved by evidence alone, values and deeply grounded beliefs affect peoples’ decision-making within these issues (Levinson, 2006; Rundgren et al., 2016).

While the utilization of such topics has shown to benefit student learning in science subjects such as biology (Klosterman and Sadler, 2010), prior studies demonstrated how teachers’ attitudes may affect their teaching approaches and knowledge about such issues (Liu et al., 2015; Plutzer et al., 2016). Because deeper values lay the foundation for attitudes (Whittaker et al., 2006), the investigation of values as appraisal dimension of anticipated enjoyment for teaching seems reasonable.

Furthermore, the subject of biology involves socioscientific issues from multiple topical domains such as ecological or health-related issues (Zeyer and Kyburz-Graber, 2012; Zeyer and Dillon, 2014). For example, researchers have already investigated teaching motivation in environmental topics such as returning wolves (Büssing et al., 2019c) or cancer education (Heuckmann and Asshoff, 2014; Heuckmann et al., 2018). This may be interesting for the investigation of differences between the relevance of universalism and benevolence as appraisal basis for anticipated enjoyment for teaching, because of their different value focus. Furthermore, we chose topics with enough variation for the variables of interest (universalism, benevolence, and psychological distance).

#### Return of Wild Wolves

For this study, we selected the return of wild wolves to Germany as a topic with a focused ecological background. After their eradication in the 19th century, wolves have migrated naturally back into parts of Europe (Chapron et al., 2014). This led to value-based conflicts with stakeholders such as farmers (Büssing et al., 2019a), who are faced with economic damages based on livestock killings (Enserink and Vogel, 2006). Aside from this economic dimension, people also fear the wolf based on deeply rooted implicit beliefs connected to the stereotype of the “Big Bad Wolf” (Jürgens and Hackett, 2017).

In biology education, the issue may be used to foster students’ understanding of ecology and biodiversity conservation, based on the discussion about the impacts of wolves on ecosystem biodiversity (Grace, 2009). Because of the economic and social dimension, the issue may also be of high interest for ESD, which aims for the integration of these domains (Leicht et al., 2018). Besides this, prior studies have shown how the personal involvement and locality of the issue may foster students’ interests and motivations when discussing this issue in schools (Hermann and Menzel, 2013b) and may also affect their proenvironmental orientations (Hermann and Menzel, 2013a).

#### Climate Change

To ensure a greater variance between the issues while controlling the domain, we selected the global environmental problem of climate change as a contrasting second topic for our investigation. Like the topic of returning wolves, climate change also involves value-based and emotionally charged dimensions and may be integrated into biology education to learn about ecosystems on a more global level (Busch and Osborne, 2014; Monroe et al., 2019). But instead of being concentrated on specific regions such as returning wolves, climate change describes a global problem (Intergovernmental Panel on Climate Change, 2014). Studies with explicit reference to psychological distance came to the same conclusions, as climate change often is experienced as a rather abstract process with a large psychological distance (McDonald et al., 2015). Because of the inherent environmental dimension in line with our first topic, we also hypothesize a positive relationship with universalism and a negative relationship with psychological distance.

#### Preimplantation Genetic Diagnosis

Besides these two environmental issues, we intentionally selected preimplantation genetic diagnosis as a third issue from a different domain to induce more variance into the data collection and to compare the role of values as antecedents also in a domain not related to environmental topics. Preimplantation genetic diagnosis refers to the usage of genetic modification methods for human embryos and constitutes a recent open societal health issue debated in science and in society (Sadler and Zeidler, 2004). Like environmental topics, contextual health issues might be used to foster student interests (Zeyer and Dillon, 2014).

## Materials and Methods

### Research Design and Participants

As we were interested in connections between specific variables, we followed a cross-sectional quantitative research design, using a paper-and-pencil questionnaire. While data from self-report questionnaires can be biased because of social desirability induced by the participants, questionnaires remain an important and efficient way to measure teaching emotions (Frenzel, 2014).

All questionnaires were distributed in July and August 2016 at four universities from northwestern, southern, and eastern Germany. We selected these locations to ensure a sufficient variance within the sample for the local teaching topic of returning wolves. The species is fairly long established in eastern Germany (since around 2000) but rather new in northwestern part of the country, and it has not yet entered southern Germany (Ansorge et al., 2010; Landesjägerschaft Niedersachsen e, 2016).

As we were investigating the anticipated enjoyment for teaching about biology topics, we surveyed only preservice teachers who were studying to become biology teachers. We ensured this by handing out the questionnaires in university lectures and courses for biology preservice teachers. Overall, 189 biology preservice teachers participated in the survey (73.5% female, age range from 19 to 50 years, mean_age_ = 23.45 years, *SD*_age_ = 3.71 years). Because of the relatively small sample size and high proportion of female preservice teachers, future studies need to expand this sample for further generalization.

In Germany, the studies for becoming a teacher are divided into bachelor’s and master’s levels of study, and prospective teachers have to decide at the start of their studies for which type of school they want to become teachers (Cortina and Thames, 2013). Within our sample, the majority of preservice teachers were still in their bachelor studies (72%), whereas the rest were in the master’s level. Because of the high proportion of bachelor students, the preservice teachers most likely had only few actual experiences with teaching. Concerning the type of school, the majority studied to become a teacher in high schools (“Gymnasium,” 54.9%), followed by secondary schools (“Hauptschule” and “Realschule,” 27.7%) and vocational schools (“Berufsschule,” 17.3%).

All procedures were in accordance with the ethical standards of the institutional and national research committees, the American Psychological Association’s Code of Conduct, and with the 1964 Declaration of Helsinki and its later amendments or comparable ethical standards. We obtained informed consent verbally and written, guaranteed anonymity, and provided information about the purpose of the study. All participants had the chance to ask questions about the questionnaire and the overall research and to decline their participation at any time.

### Measures

#### General Approach

The questionnaire started with an introductory text explaining the purpose and aims of the study, followed by questions about demographic data. After this general part, three context-specific parts including the presented constructs completed the questionnaire. As the questionnaire was part of the publication-based dissertation of the first author, it included several other scales that are not part of this report. Further information may be obtained from the published dissertation (Büssing, 2018).

Each of the more specific parts started with a short description of the contextual background information about these socioscientific issues to ensure a common understanding. The order of the contexts within the questionnaires was randomized using different printed versions with all possible combinations of contexts to rule out any order effects. Participants needed approximately 25 min to complete the questionnaires.

All latent variables were measured by multiple indicators to enhance the validity of the constructs. All items were measured on a six-point Likert scale, ranging from 1 (do not agree at all) to 6 (agree completely), and were worded as statements (Bryman, 2008). The German and English versions of all scales can be found in the Supplementary Material.

#### Demographic Data

For demographic data, we asked for the age, gender, and intended degree of participants. While age and the intended degree were open questions, gender was asked in a closed format and coded with 1 (female) and 2 (male). The intended degree was used only to ensure the affiliation to the intended sample and was not further analyzed. We also excluded this variable in the uploaded dataset to ensure the anonymity of our participants.

#### Universal Values

We used the corresponding subscales of the German 40-item Portrait Value Questionnaire (PVQ-40) to measure the value dimensions of universalism and benevolence (Schmidt et al., 2007). These scales are well-established and validated within varied sample populations and countries (Cieciuch and Davidov, 2012) and include six items for universalism and four items for benevolence.

One sample item from the universalism scale is “I strongly believe that people should care for nature. Looking after the environment is important to me,” and from the benevolence scale, “It is very important for me to help the people around me. I want to care for their well-being.”

#### Psychological Distance

As described above, psychological distance comprised the four dimensions of temporal, spatial, social, and hypothetical distance (Liberman and Trope, 2014). Because of the lack of a standardized measure, we developed our own scale based on existing studies by constructing an overall scale with one item per dimension of psychological distance, which resulted in four items for each context and 12 items for all three contexts (Büssing et al., 2019c).

One sample item is “I am personally concerned by returning wolves.” We asked for the concern about the selected issues and not the perceived distance to ensure that all participants have a similar understanding, as people experience distance as closeness (Trope and Liberman, 2010). We calculated the means of all items to construct the final scales for each topic. Before this, all items were coded reversely, based on the theoretical definition of psychological distance as distance and not proximity.

#### Anticipated Enjoyment for Teaching

For the anticipated enjoyment for teaching, we used the German version of the established and validated general Teacher Emotion Scale (Frenzel et al., 2016). This scale measures the general enjoyment for teaching, but as this does not specify items for particular topics, we adapted the items to capture the anticipated enjoyment for teaching the specific topics by directly adding the respective topics to the items. Overall, the original scale comprised four items for measuring enjoyment for teaching. As the scale needed to be applied to all three topics, the scales for measuring anticipated enjoyment for teaching compromised 12 items in total of the questionnaire.

To ensure that participants responded with their anticipated teaching, we added a prompt prior to the items. The prompt said: “If you think about your future time as a teacher, how strongly would you confirm with the following statements?” This is also visible in Table 1 besides the wording of all items.

**TABLE 1:** Standardized factor loadings (λ) for the anticipated enjoyment for teaching from confirmatory factor analysis (CFA) for the theoretical three-factor structure and alternative two-factor and one-factor structure (displayed in Figure 2) with fit indices.

	Standardized factor loading		
Item	Three-factor	Two-factor	One-factor
If you think about your future time as a teacher, how strongly would you confirm with the following statements?			
I generally enjoy teaching the topic of the return of the wolf. (WEMOJOY1)	0.846	0.845	0.844
I generally have so much fun teaching about the topic of the return of the wolf that I gladly prepare and teach my lessons. (WEMOJOY2)	0.872	0.878	0.879
I often have reasons to be happy while I teach about the return of the wolf. (WEMOJOY3)	0.798	0.790	0.787
I generally teach about the return of the wolf with enthusiasm. (WEMOJOY4)	0.945	0.930	0.924
I generally enjoy teaching the topic of climate change. (CEMOJOY1)	0.827	0.431	0.439
I generally have so much fun teaching about the topic of climate change that I gladly prepare and teach my lessons. (CEMOJOY2)	0.907	0.449	0.458
I often have reasons to be happy while I teach about the topic of climate change. (CEMOJOY3)	0.555	0.363	0.369
I generally teach about the topic of climate change with enthusiasm. (CEMOJOY4)	0.935	0.435	0.443
I generally enjoy teaching the topic of preimplantation genetic diagnosis. (PEMOJOY1)	0.883	0.878	0.121^n.s.^
I generally have so much fun teaching about the topic of preimplantation genetic diagnosis that I gladly prepare and teach my lessons. (PEMOJOY2)	0.755	0.757	0.077^n.s.^
I often have reasons to be happy while I teach about the topic of preimplantation genetic diagnosis. (PEMOJOY3)	0.595	0.597	0.130^n.s.^
I generally teach about the topic of preimplantation genetic diagnosis with enthusiasm. (PEMOJOY4)	0.885	0.886	0.171^n.s.^
**Model fit**			
χ^2^ test (degrees of freedom)	94.533 (51)	411.045 (53)	674.484 (54)
Robust comparative fit index	0.96	0.69	0.45
Robust root mean square error of approximation	0.08	0.22	0.29
Standardized root mean square residual	0.05	0.17	0.23

*n.s. = not significant loading of the item. The definitions in brackets show the name of the respective items and were not part of the questionnaire.*

### Statistical Analysis

#### General Approach

**FIGURE 2:**
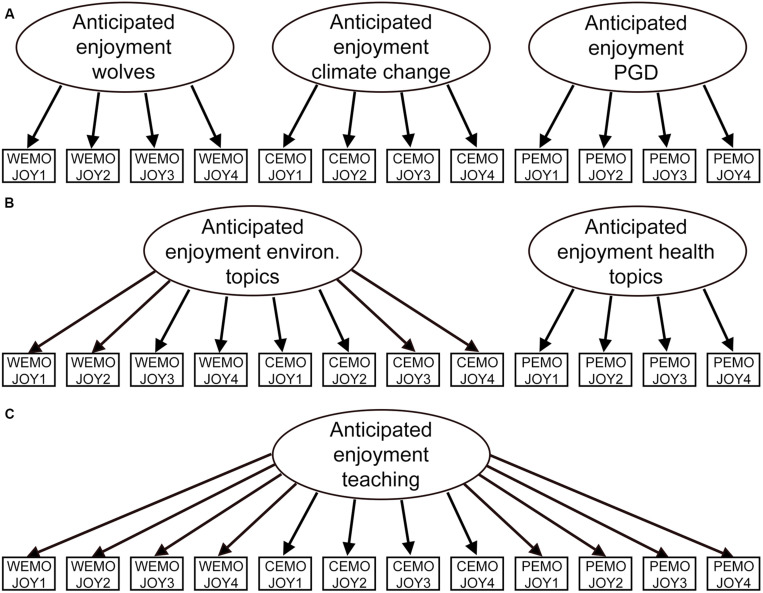
Illustration of the applied measurement models for the confirmatory factor analysis (CFA) with the theoretical three-factor **(A)** and two alternative models with two factors **(B)** and one factor **(C)**.

We began investigation of the measurement results with confirmatory factor analyses (CFAs) and Cronbach α. As we had overall three scales (universal values, psychological distance, and anticipated enjoyment for teaching) related to three different topics, we estimated three different models for each measure. First, we calculated a theoretical model with one factor per topic, resulting in a three-factor structure (example A in Figure 2). Following this, we calculated a first alternative model, which included two factors that concentrated on the topical domain (environmental and health topics; example B in Figure 2). Finally, we calculated a second alternative model with a one-factor structure (example C in Figure 2). With this approach, we secured a sufficient discriminant validity of our scales (Brown, 2015). Figure 2 illustrates the estimated measurement models and their underlying items for the example of anticipated enjoyment for teaching.

In accordance with Brown (2015), we evaluated model fit with the χ^2^ test statistics (the lower the better), robust comparative fit index (CFI; ≥0.95), robust root mean square error of approximation (RMSEA; ≤0.08), and standardized root mean square residual (SRMR; ≤0.08). The threshold for a sufficient loading of individual items was set to 0.40 (Field, 2019).

Because of the small sample size, we were not able to calculate one large CFA simultaneously including all factors, as such a model would include too many free parameters, and the recommended ratio of free parameters to participants (1:5) would not be fulfilled (Brown, 2015). This could be an objective for future studies with more participants to generalize our measurement results to other samples, especially also with in-service teachers.

Based on the results of the CFA, we constructed the scales from the resulting items. We proceeded by investigating the bivariate correlations and descriptive statistics. Following this, we inspected the corresponding theoretical hypotheses. For investigating the antecedents of anticipated enjoyment for teaching, we used regressions and followed a three-step approach. First, we investigated the predictive ability of the values and psychological distance with models that included only these variables and then continued by adding demographic control variables in a second step. In particular, we included age and gender as control variables into our analyses based on their effects in prior studies (Lohbeck et al., 2018). Finally, we calculated an overall path model to account for the connections between all intrapersonal variables.

As the skewness and kurtosis indicated a skew for some variables, we used robust statistical methods, based on Field and Wilcox (2017). This included robust regressions and correlations (Spearman ρ was used as correlation coefficient). For the final path model, we used a robust maximum likelihood estimator based on lavaan (Rosseel, 2012). All calculations were done in R Studio version 1.1.456 running R version 3.5.1 (R Core Team, 2013). The script and data for a replication of our analysis are available in the Supplementary Material of the article.

#### Measurement Results

The initial measurement model for the universal values showed no sufficient fit to the data [χ^2^(34) = 84.335, CFI = 0.88, RMSEA = 0.09, SRMR = 0.09]. While prior studies had already indicated problems with the universalism scale based on its large spectrum of contents (environment and people), we inspected the factor loadings of the scales to modify the model based on theoretical assumptions (Brown, 2015). We excluded one item from the universalism scale (PVQ02), which showed a too close relationship to the factor of benevolence. The modified model then achieved a good fit to the data [χ^2^(26) = 39.810, CFI = 0.96, RMSEA = 0.06, SRMR = 0.06]. The one-factor model showed the worst fit to the data [χ^2^(35) = 151.177, CFI = 0.72, RMSEA = 0.14, SRMR = 0.10].

For the psychological distance scale, the theoretical model achieved a sufficient fit to the data [χ^2^(51) = 79.239, CFI = 0.97, RMSEA = 0.06, SRMR = 0.06). The alternative models with two factors [χ^2^(53) = 381.211, CFI = 0.61, RMSEA = 0.20, SRMR = 0.18] and one factor [χ^2^(54) = 564.169, CFI = 0.36, RMSEA = 0.25, SRMR = 0.21] showed no fit to the data.

While more information for the CFAs for the universal values and psychological distance may be obtained from the Supplementary Material, the results for the anticipated enjoyment for teaching scale are presented in Table 1 as the first part of the results of this article. As a sufficient fit to the data and sufficient values of Cronbach α were achieved for all variables, we accepted the models with the given modification and continued with further analysis.

## Results

### Factor Analysis of Topic-Specific Anticipated Enjoyment for Teaching

The results of the CFAs for the anticipated enjoyment for teaching are displayed in Table 1. Overall, the theoretical model with a three-factor structure showed the best fit to the data [χ^2^(51) = 94.533, CFI = 0.96, RMSEA = 0.08, SRMR = 0.05]. The alternative models with two factors [χ^2^(53) = 411.945, CFI = 0.69, RMSEA = 0.22, SRMR = 0.17] and one factor [χ^2^(54) = 674.484, CFI = 0.45, RMSEA = 0.29, SRMR = 0.23] showed no fit to the data.

Besides these fit criteria, we found the most consistent factor loadings in the model with the three-factor structure, as all loadings were above the threshold of 0.40. Interestingly, the loadings in the one-factor model resembled the content domains, as the items for the returning-wolves topic ranged at high levels (λ_WOLF_ = 0.924–0.787), the loadings for the topic of climate change were lower around the threshold of 0.40 (λ_CC_ = 0.458–0.369), and the loadings for the preimplantation genetic diagnosis were only marginal (λ_PGD_ = 0.171–0.077).

Overall, these are positive indications for the fit of the topic-specific model to the data. Therefore, we proceeded with the further analysis.

### Intercorrelations and Descriptive Statistics

As described in Table 2, we found several intercorrelations between the study variables. Because of the multiple significance tests, we report the adjusted *p*-values; if the values were greater than 0.001, we give the exact value. Both the adjusted and the non-adjusted *p*-values are reported only if they differ for the results of significance tests.

**TABLE 2:** Overview of the correlations (Spearman ρ) and descriptive statistics for the measured variables with adjusted *p*-values above the diagonal and unadjusted values under the diagonal.

	1	2	3	4	5	6	7	8	9	10
(1) Age	–	0.10	0.23*	0.06	0.08	–0.03	0.04	0.24*	0.07	0.00
(2) Gender	0.10	–	–0.02	–0.11	–0.15	–0.18	–0.08	0.27*	0.23*	–0.20
(3) Universalism	0.23***	–0.02	–	0.39***	–0.11	−0.36***	–0.00	0.29***	0.25*	0.04
(4) Benevolence	0.06	–0.11	0.39***	–	–0.13	–0.19	–0.08	0.13	0.23*	0.23*
(5) PD_WOLF_	0.08	−0.15*	–0.11	–0.13	–	0.10	0.24*	−0.25*	–0.16	0.01
(6) PD_CC_	–0.03	−0.18*	−0.36***	−0.19*	0.10	–	0.04	−0.25*	−0.35***	–0.11
(7) PD_PGD_	0.04	–0.08	–0.00	–0.08	0.24***	0.04	–	–0.04	–0.01	–0.21
(8) Enjoyment_WOLF_	0.24***	0.27***	0.29***	0.13	−0.25***	−0.25***	–0.04	–	0.40***	0.08
(9) Enjoyment_CC_	0.07	0.23***	0.25***	0.23***	−0.16*	−0.35***	–0.01	0.40***	–	0.24*
(10) Enjoyment_PGD_	0.00	−0.20*	0.04	0.23***	0.01	–0.11	−0.21***	0.08	0.24***	–
Number of Items	1	1	5	4	4	4	4	4	4	4
Mean	23.45	–	5.07	4.99	3.68	2.10	4.81	3.91	4.06	4.00
Standard deviation	3.71	–	0.57	0.55	1.04	0.88	0.86	0.97	0.89	0.84
Median	23.00	–	5.00	5.00	3.75	2.00	5.00	4.00	4.00	4.00
Skewness	–	–	–0.34	–0.26	–0.28	0.51	–0.88	–0.46	–0.15	–0.46
Kurtosis	–	–	–0.58	–0.11	–0.51	–0.47	0.37	0.30	0.41	0.56
Cronbach α	–	–	0.74	0.66	0.83	0.85	0.74	0.92	0.87	0.85

* = p < 0.05, *** = p < 0.001. Gender was coded with female (1) and male (2). WOLF = Return of the wolf, CC = Climate change, PGD = Pre-implantation genetic diagnosis.

We found the largest correlation with a medium effect size for the anticipated enjoyment for teaching about returning wolves and climate change (*r* = 0.40, *p* < 0.001) and a correlation with a smaller effect size for the anticipated enjoyment for teaching about climate change and preimplantation genetic diagnosis (*r* = 0.24, *p* = 0.04). There was no correlation between the anticipated enjoyment for teaching about returning wolves and preimplantation genetic diagnosis (*r* = 0.08, *p* = 1.00).

Besides the intercorrelations of the anticipated enjoyment for teaching the respective topics, we also found connections of the universal values of universalism and benevolence with the anticipated enjoyment for teaching about the topics. While universalism correlated with the anticipated enjoyment for teaching about returning wolves (*r* = 0.29, *p* < 0.001) and the anticipated enjoyment for teaching about climate change (*r* = 0.25, *p* = 0.02), there was no correlation of universalism with the anticipated enjoyment for teaching about preimplantation genetic diagnosis (*r* = 0.04, *p* = 1.00). But anticipated enjoyment for teaching about preimplantation genetic diagnosis was correlated with benevolence, even if the corrected *p*-value was not significant (*r* = 0.23, *p*_NOADJ_ < 0.001, *p*_ADJ_ = 0.05). Furthermore, there were also correlations between benevolence and the anticipated enjoyment for teaching about climate change, even if the *p*-value again showed no significant relation after its adjustment (*r* = 0.23, *p*_NOADJ_ < 0.001, *p*_ADJ_ = 0.05). There was no correlation for the value of benevolence and the anticipated enjoyment for teaching about the topic of returning wolves (*r* = 0.13, *p* = 1.00).

Concerning the psychological distances toward the issues, we found psychological distance toward all issues correlated with the anticipated enjoyment for teaching about the respective issue. The strongest connection was found between the psychological distance toward climate change and anticipated enjoyment for teaching about the issue (*r* = −0.35, *p* < 0.001), followed by the psychological distance toward returning wolves and the anticipated enjoyment for teaching about the issue (*r* = −0.25, *p* = 0.02). Finally, the psychological distance toward preimplantation genetic diagnosis also correlated with the anticipated enjoyment for teaching about the topic, but only for the non-adjusted *p*-value (*r* = −0.21, *p*_NOADJ_ < 0.001, *p*_ADJ_ = 1.00).

Concerning the demographic variables, age was positively correlated with the anticipated enjoyment for teaching about returning wolves (*r* = 0.24, *p* = 0.03), but not with the anticipated enjoyment for teaching about the other issues. This means that older preservice teachers showed a higher anticipated enjoyment for teaching about wolves. This effect may be due to the higher universalistic orientation of older people, which was indicated by the correlation between universalism and age (*r* = 0.23, *p* = 0.04). But because of the small variance of age in our sample, this result should be generalized cautiously, as we will discuss later.

Gender was consistently correlated with the anticipated enjoyment for teaching about all three topics, even though the direction of this effect differed between them. The variable was positively correlated with the anticipated enjoyment for teaching about returning wolves (*r* = 0.27, *p* = 0.01) and climate change (*r* = 0.23, *p*_NOADJ_ < 0.001, *p*_ADJ_ = 0.05), even though this second correlation, again, was only near significance when adjusted for multiple tests. Similarly, although the correlation between gender and the anticipated enjoyment for teaching about preimplementation genetic diagnosis also reached only significant results without the adjustment of the *p*-values, it was negatively correlated (*r* = −0.20, *p*_NOADJ_ = 0.01, *p*_ADJ_ = 0.17). That is, female preservice teachers showed a higher anticipated enjoyment for teaching about the preimplantation genetic diagnosis and male preservice teachers about climate change and the return of wild wolves.

Table 2 also illustrates the descriptive statistics of the variables. Concerning the anticipated enjoyment for teaching, distributions differed only marginally between the issues. The highest anticipated enjoyment was reported for teaching about climate change (mean = 4.06, *SD* = 0.89, median = 4.00), followed by the anticipated enjoyment for teaching about preimplantation genetic diagnosis (mean = 4.00, *SD* = 0.84, median = 4.00). The preservice teachers reported the smallest anticipated enjoyment for teaching about returning wolves (mean = 3.91, *SD* = 0.97, median = 4.00). Because of these differences being small, we stepped away from computing difference tests and continued with investigating the antecedents of the anticipated enjoyment for teaching.

### Prediction of Anticipated Enjoyment for Teaching

#### Robust Regression Analyses

As shown in the first step of the robust regression analysis displayed in Table 3, universalism was a predictor for the anticipated enjoyment for teaching the topic of the return of wild wolves (β = 0.48, *p* < 0.001), but not for the other topics of climate change (β = 0.15, *p* = 0.17), and preimplantation genetic diagnosis (β = −0.07, *p* = 0.57). Similarly, benevolence predicted only the anticipated enjoyment for teaching about the topic of preimplantation diagnosis (β = 0.33, *p* < 0.001), but not for the anticipated enjoyment for teaching about the return of wolves (β = 0.00, *p* = 0.98) or about climate change (β = 0.21, *p* > 0.08). Psychological distance was the only predictor for all three topics: the return of wolves (β = −0.23, *p* < 0.001), climate change (β = −0.29, *p* < 0.001), and preimplantation genetic diagnosis (β = −0.17, *p* = 0.01).

**TABLE 3:** Standardized robust regression results (β) with standard error (SE) for the prediction of anticipated enjoyment for teaching about the topics of returning wolves (wolf), climate change, and preimplantation genetic diagnosis (PGD).

	Wolf	Climate change	PGD
	β (*SE*)	β (*SE*)	β (*SE*)
**Step 1**			
Intercept	2.37* (0.96)	2.87*** (0.80)	3.56*** (0.68)
Universalism	0.48*** (0.14)	0.15 (0.11)	−0.07 (0.13)
Benevolence	−0.00 (0.16)	0.21 (0.12)	0.33** (0.11)
Psychological distance	−0.23** (0.07)	−0.29** (0.09)	−0.17** (0.06)
Adjusted R^2^	0.14	0.15	0.07
**Step 2**			
Intercept	0.84 (0.97)	1.76 (0.87)	4.29*** (0.70)
Age	0.03 (0.02)	0.00 (0.01)	−0.00 (0.01)
Gender	0.45*** (0.13)	0.42** (0.14)	−0.38** (0.13)
Universalism	0.44** (0.13)	0.17 (0.09)	−0.07 (0.12)
Benevolence	0.07 (0.15)	0.27* (0.12)	0.30** (0.11)
Psychological distance	−0.18** (0.07)	−0.23** (0.09)	−0.19** (0.06)
Adjusted *R*^2^	0.19	0.18	0.11

* = p < 0.05, ** = p < 0.01, *** = p < 0.001. R^2^ = Explained variance within the respective dependent variable. Gender was coded as female (1) and male (2).

In the second regression step, we included age and gender as control variables. While the regression coefficients slightly decreased, the significant predictors kept their predictive ability for the anticipated enjoyment for teaching, even when gender emerged as strongest predictor within every topic. Overall, anticipated enjoyment for teaching about the return of wolves was predicted by universalism (β = 0.44, *p* < 0.001), psychological distance (β = −0.18, *p* = 0.01), and gender (β = 0.45, *p* < 0.001). Anticipated enjoyment for teaching about climate change was predicted by benevolence (β = 0.27, *p* = 0.02), psychological distance (β = −0.23, *p* = 0.01), and gender (β = 0.42, *p* < 0.001). Finally, the anticipated enjoyment for teaching about preimplantation genetic diagnosis was still predicted by benevolence (β = 0.30, *p* = 0.01), psychological distance (β = −0.19, *p* < 0.001), and gender (β = −0.38, *p* < 0.001).

Similar to the correlations, female preservice teachers showed a smaller anticipated enjoyment for teaching about the return of wolves and about climate change, because of the positive predictive effect of gender [female coded as (1) and male coded as (2)]. For the anticipated enjoyment for teaching about preimplantation genetic diagnosis, this effect reversed, as gender was a negative predictor; that is, female participants showed an increased anticipated enjoyment for teaching about this topic than male participants.

The explained variance differed between the models and was increased by the second regression step. While the first step explained approximately 14% of the variance in the anticipated enjoyment for teaching about the return of the wolf (adjusted *R*^2^ = 0.14), the second step explained approximately 19% (adjusted *R*^2^ = 0.19). Similarly, the first step for the regression of anticipated enjoyment for teaching explained 15% (adjusted *R*^2^ = 0.15) and the second 18% of the variance in the dependent variable (adjusted *R*^2^ = 0.18). Overall, the selected predictors explained the least variance for the anticipated enjoyment for teaching about preimplantation genetic diagnosis, by explaining only 7% (adjusted *R*^2^ = 0.07) in the first and 11% (adjusted *R*^2^ = 0.11) of the variance in the second regression step.

#### Path Model

Finally, we calculated a path model to account for the intrapersonal structure between all variables. While the path model showed a sufficient fit to the data [χ^2^(6) = 7.518, CFI = 0.99, RMSEA = 0.04, SRMR = 0.03], the predictive effects from the regressions were mostly replicated, except for universalism, which now also significantly predicted the anticipated enjoyment for teaching about climate change.

As displayed in Table 4, universalism predicted the anticipated enjoyment for teaching about returning wolves (β = 0.26, *p* < 0.001) and the anticipated enjoyment for teaching about climate change (β = 0.14, *p* = 0.03). Benevolence predicted only the anticipated enjoyment for teaching about preimplantation genetic diagnosis (β = 0.18, *p* = 0.01). Psychological distance and gender predicted all dependent variables, consistent with the prior single-regression models, even though gender was no longer the strongest predictor within every topic. The strongest predictor within the topic of the return of wolves was universalism, and within the topic of preimplantation genetic diagnosis, psychological distance.

**TABLE 4:** Standardized regression results (β) with standard error (SE) from the path model for the regressions of anticipated enjoyment for teaching about returning wolves (Wolf), climate change, and preimplantation genetic diagnosis (PGD).

	Wolf	Climate change	PGD
Predictors	β (*SE*)	β (*SE*)	β (*SE*)
Age	0.09 (0.02)	−0.01 (0.01)	−0.00 (0.01)
Gender	0.22*** (0.13)	0.23** (0.13)	−0.20** (0.13)
Universalism	0.26*** (0.12)	0.14* (0.10)	−0.01 (0.12)
Benevolence	0.02 (0.15)	0.13 (0.13)	0.18* (0.11)
Psychological distance	−0.20** (0.06)	−0.16* (0.07)	−0.21*** (0.06)
Adjusted *R*^2^	0.21	0.15	0.12
**Model fit**			
χ^2^ (*df*)	χ^2^(6) = 7.518		
CFI	0.99		
RMSEA	0.04		
SRMR	0.03		

* = p < 0.05, ** = p < 0.01, *** = p < 0.001. R^2^ = Explained variance within the respective dependent variable. Gender was coded as female (1) and male (2). χ^2^ = Chi-square test, df = degrees of freedom, CFI = robust comparative fit index, RMSEA = robust Root Mean Square Error of Approximation, SRMR = Standardized Root Mean Square Residual.

Based on the integration of all variables as predictors, the explained variance increased for the anticipated enjoyment for teaching about returning wolves (adjusted *R*^2^ = 0.21), climate change (adjusted *R*^2^ = 0.15), and the preimplantation genetic diagnosis (adjusted *R*^2^ = 0.12).

## Discussion

### Topic Specificity of Anticipated Enjoyment for Teaching

Concerning our first hypothesis (H_1_), we found the best fit of a topic-specific model to the data for the anticipated enjoyment for teaching, different correlations between the selected topics, and differences between the variables that predicted the dependent variables. Therefore, we believe there may be several indications of the suitability of a topic-specific approach to anticipated enjoyment for teaching in our study.

In prior research, the specificity of enjoyment for teaching has already been discussed and empirically investigated. For example, Frenzel et al. (2016) advocated for a more specific approach to teaching emotions and tested the applicability of general and subject-specific scales of teacher emotions. This is in line with two prior studies that showed how a large proportion of variation of teaching emotions is explainable on the contextual level (Frenzel et al., 2015).

In addition to these reasons for a topic-specific approach to emotions, our study also illustrated reasons to disagree with such an approach to teaching emotions. The major reason would be the only marginal differences in the distributions of the different topics, which also showed a negative skew. At the moment, we are not able to definitively explain this result, even if it may be likely due to the nature of our sample. More particularly, preservice teachers may have had only few or no experience with teaching and therefore few conceptions about what it means to teach specific topics. This may have led to only a small variation between the topics. Again, future studies are needed to investigate if in-service teachers may show more variation in their enjoyment for teaching about specific topics.

Nonetheless, a topic-specific approach to emotions enables such a consideration, even when further work is needed to generalize these results. This potential was also visible in the differentiation of the antecedents for anticipated enjoyment for teaching between the three selected topics. Such a study would only be possible when emotions are viewed from a topic-specific perspective.

### Antecedents of Anticipated Enjoyment for Teaching

#### Universal Values

As the results of the present study show, both universal values we investigated were topical predictors of anticipated enjoyment for teaching in the selected topics.

We found universalism as a value for the preservation and the well-being of all people and for nature as a predictor of enjoyment for teaching about the return of wolves, which supports our hypotheses (H_2_). At the same time, universalism was also correlated with the enjoyment for teaching about climate change, but no predictor in the robust regressions. However, in the path model, it gained predictive abilities again.

Similarly, benevolence predicted only the enjoyment for teaching about the health topic of preimplantation genetic diagnosis in line with our third hypothesis (H_3_), while it was also correlated to the anticipated enjoyment for teaching about climate change and was a predictor for this topic in the robust regressions. The predictive effect was not visible in the path model. We believe this result may be explainable by the contextual background of the topic of climate change. While the return of wolves stems from a strict environmental background, the topic of climate change obviously includes a strong personal focus. This was illustrated by the correlations, as the anticipated enjoyment for teaching about climate change was correlated with the anticipated enjoyment for teaching both other topics, but the anticipated enjoyment between the other topics was not correlated.

While prior studies about teaching emotions most often considered the appraisal of emotions based only on student behavior (Frenzel, 2014; Becker et al., 2015), the predictive abilities of universal values for anticipated enjoyment for teaching illustrate how further topical variables may play a role in underlying appraisal processes. This adds important knowledge about the antecedents of anticipated enjoyment for teaching and assumes a more positive emotional view of teachers when they teach about topics consistent with their own value structures, as has already been shown for the topic of inclusive teaching (Büssing et al., 2019b).

Hence, teachers who are faced with teaching about topics with a higher obligation such as ESD may appraise teaching about these issues more positively when they possess a corresponding underlying value structure. This result is in line with prior research, which found values and other personality variables as a contributor to in-service teachers’ motivations to teach about the topic of climate change (McNeal et al., 2017).

For the case of teaching emotions, this implies severe differences in emotional experience when teaching about positively versus negatively valued topics. Because of the nature of the present study, we are unable to determine if the underlying value structure may lead to an extended commitment over time because of more idealism for teaching about the topic, because the reverse connection may also be plausible. Our sample may also differ from in-service teachers because of the transformative role of integrating into existing external structures such as a new school, which may also change preservice teachers’ behavior and value structures (Rust, 1994). Therefore, a future study should adopt the approach of selecting universal values as predictors of the anticipated enjoyment for teaching about specific topics in combination with other variables, such as anger about student behavior or perceived behavioral control toward teaching. This would allow one to assess the role of the more topic-specific factors that have been found in our study.

But while our results should be generalized only cautiously to in-service teachers, they still constitute a first investigation of the effects of values as contextual determinants for the appraisal of anticipated enjoyment for teaching. As we will discuss later, teacher preparation courses should implement professional development activities to reflect on and process existing value structures and, if necessary, try to utilize deep learning experiences such as transformative learning to develop required understandings (Mezirow, 2009).

#### Psychological Distance Toward Issues

Psychological distance emerged as the second major predictor of anticipated enjoyment for teaching because of its predictive ability for all three dependent variables. This is in line with our hypotheses (H_4_) and implies a higher anticipated enjoyment for teaching psychologically close contexts. As the integration of this variable into education research is rather new, we believe this finding to be considered as of a more explorative nature, although this is the second such study with these effects (Büssing et al., 2019c).

The predictive ability in the regressions makes sense based on the underlying psychological processes in line with prior research about the relationships between psychological distance and emotions (Van Boven et al., 2010). When teachers feel close to a specific issue, they may rate this topic as more personally important to them, which also could indicate a higher personal relevance (Stuckey et al., 2013). Furthermore, teachers may have more direct experiences of close issues in comparison to distant issues they might barely have heard of. This could also affect knowledge structures, which we have not integrated in the present study, but which might be interesting for further investigation.

Further research is also needed to understand the differences between the psychological distances for the issues. Our results partly contradicted prior studies, as climate change has often been described as a relatively distant event (Hufnagel, 2015), but the preservice teachers in our sample reported feeling only a very small psychological distance to the issue. We believe this result might be explainable by the most recent developments around the issue of climate change. For example, the election of Donald Trump as the President of the United States has weakened the global aspirations to fight human-caused climate change, which may have increased the preservice teachers’ awareness of the issue, coupled with their perceived obligation to protect the planet through teaching about the issue, as, for example, a prior study demonstrated connections between awareness and general protection motivations for this particular issue (Dal et al., 2015). This interpretation would be consistent with the highest anticipation of enjoyment for teaching about climate change we found here. Even if these differences were rather small and not explicitly tested as part of our article, further studies should investigate how teachers who are concerned of specific topics may differ in their psychological distance to other, more general samples based on evaluations of distance.

#### Gender and Anticipated Enjoyment for Teaching

As described in our results, we found gender to be a strong predictor of the anticipated enjoyment for teaching about the respective topics. Overall, male participants showed a higher anticipated enjoyment for teaching about the topics of the return of wolves and climate change, whereas female preservice teachers anticipated more enjoyment for teaching about preimplantation genetic diagnosis. These results are in line with general psychological studies about gender as a determinant of emotional experience for a wide variety of behaviors (Brody and Hall, 2008), but are rather inconsistent with prior studies about teaching emotions. While some studies found gender to be connected to a higher elicitation of anxiety (Lohbeck et al., 2018), other studies showed no differences between male and female teachers (Sutton and Wheatley, 2003; Frenzel et al., 2020). While many studies disregarded the content dimension for the measurement of emotions and concentrated on general classroom processes, the explicit integration of this topic-specific dimension is the first major difference between our study and previous approaches to teaching emotions and may explain some of these inconsistencies.

Based on content-related dimensions, we found coherent differences between female and male preservice teachers, as male teachers may show higher anticipated enjoyment for teaching about controversial issues based on their higher elicitation of positive emotions (Burke, 2015; Lohbeck et al., 2018). This effect reversed for the topic of preimplantation genetic diagnosis. The nature of this issue in connection to pregnancy may explain this result, as female teachers may be more interested in the required background knowledge about the topic (Krapp and Prenzel, 2011). But of course there are also several other factors to consider such as cultural norms, which is why these explanations remain preliminary, and it is also because of the quantitative nature of the current study. Future studies should pay attention to gender differences between female and male preservice teachers when including topic-specific dimensions.

## Implications

### Affectively Oriented and Topic-Specific Teacher Professional Development

In the presented and discussed connections between the anticipated enjoyment for teaching and both universal values, psychological distance illustrates how topical variables such as values may affect appraisal processes for positive emotions. As these underlying beliefs are of particular relevance to socioscientific issues (Heuckmann et al., 2018), future studies should further integrate these personality-related results into teacher professional development. Similarly, we found psychological distance as the second major contributor to anticipated enjoyment for teaching about the selected issues.

Based on our results, teacher educators should develop an awareness of motivational differences between issues, because teacher professional development activities should ideally engage teachers instead of only providing new information (Korthagen, 2017). The investigated value dimensions of universalism and benevolence have been demonstrated to be relevant to deeper identity structures within teachers, which may be addressed in teacher professional development (Day and Leitch, 2001; Korthagen, 2004; Mahler et al., 2017). Such an integration may be difficult because of the long-term stability of such values (Schwartz, 1994; Whittaker et al., 2006), which is why suitable ways of fostering the development of these affective dimensions of teaching competency in teacher education are needed. Some studies found real-world experiences and other affectively oriented learning activities to be suitable in addressing these personality variables (Molderez and Fonseca, 2018). Besides values, also other personality variables such as nature relatedness or environmental concern may be of relevance at least for teaching enjoyment about environmental topics (Weber et al., 2020).

Besides addressing underlying personality structures, professional development activities should also be planned in a topic-specific manner, as participants may strongly differ in their psychological distance toward specific instructional contents, and as our results showed, this may differentially affect their anticipated enjoyment for teaching. Finally, the predictive effects of psychological distance may be utilized either in targeted interventions or in the selection of appropriate issues for teacher education. Concerning targeted interventions, prior empirical studies showed decreases in psychological distance as a way to foster proenvironmental action (Jones et al., 2017). In a similar manner, teacher educators could think about integrating a decrease in psychological distance to relevant socioscientific issues into their teacher professional development activities to foster (preservice) teachers’ motivation. Of course, the selection of educational contents is a complex process that is bound to many (and potentially conflicting) contextual and normative considerations, but the active engagement of preservice teachers with the respective material is a central goal of professional development (Korthagen, 2017).

Finally, the results may be viewed as a counterweight to prior approaches of professional competence because of their close concentration on cognitive outcomes such as knowledge (for example Kunter et al., 2013 or Großschedl et al., 2015). Of course, teachers require a certain degree of knowledge competence to explain contents to students, but based on the functions of values and beliefs as filters of teachers’ behavior (Fives and Buehl, 2012; Raveendran and Chunawala, 2015), teachers may not be able to transfer these cognitive and conscious competencies into engaging and effective practice if topics contrast their value structures. Furthermore, the digital age may fundamentally change the conception of knowledge, which is why value and belief structures may gain increasing weight in the upcoming years (Plutzer et al., 2016). These questions should be further investigated in future field studies of practical teaching.

### Conclusion and Outlook

Based on the results from our cross-sectional study, we found psychological distance and gender as general antecedents and universal values as topic-specific antecedents of anticipated enjoyment for teaching. While these results should be considered in contemporary teacher professional development, we believe them to be only the first step toward investigating the relevance of further contextual appraisal dimensions of teaching emotions. A specific problem is the nature of our study, which mainly followed a trait-based approach to emotions and only assessed the anticipated enjoyment for teaching of preservice teachers (Frenzel et al., 2016).

**FIGURE 3:**
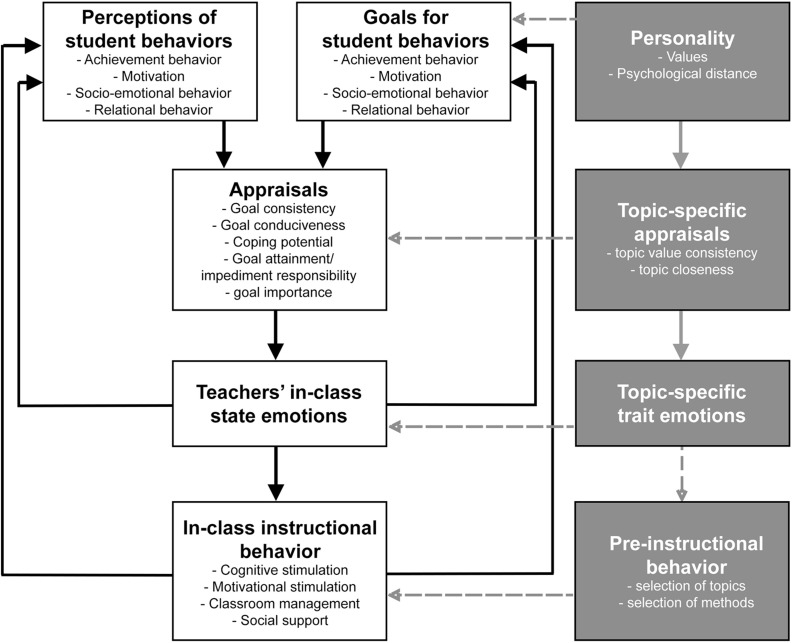
Overview of the reciprocal model on causes and effects of teacher emotions (adapted from Frenzel, 2014) in white rectangles and additional relevant variables in the gray rectangles. Black arrows stem from the original model, gray arrows show the connections investigated in this manuscript, and dashed arrows indicate possible connections that may be interesting for further studies.

As a next step, the integration of classroom field data would enable a deeper look into the practical usefulness of our results. This could be underpinned by the integration of other measures of emotional experience, such as physiological or expressiveness measures (Tobin et al., 2016). Concerning the role of personality and the variables of universal values and psychological distance for the reciprocal model of teaching emotions, we believe the topic-specific variables may play a role, even if this was not part of our study. As displayed in Figure 3, the investigated personality variables (e.g., values and psychological distance) may serve as topic-specific appraisal dimensions, demonstrating connections to topic-specific (trait) emotions. Based on this, many other connections may be plausible. For example, personality may also affect the goals for student behaviors, topic-specific appraisal may affect the student related appraisal dimensions, or topic-specific trait emotions may affect in-class state emotions. This needs to be elaborated in further studies.

These further studies may lay the foundation for better understanding of emotions in classrooms, which is particularly necessary in these times of post-truth (Peters, 2017). While our study illustrated this for the emotions toward teaching about selected topics, the results may also be reflected in further educational research about socioscientific issues and advance teacher emotion theory.

## Data Availability Statement

All datasets generated for this study are included in the article/Supplementary Material.

## Ethics Statement

Ethical review and approval was not required for the study on human participants in accordance with the local legislation and institutional requirements. The patients/participants provided their written informed consent to participate in this study.

## Author Contributions

AB conceptualized and designed the study, performed the statistical analyses, and wrote the first draft of the manuscript. JD performed the investigation and gathered the data. AB and SM reviewed and edited the manuscript. All authors contributed to the article and approved the submitted version.

## Conflict of Interest

The authors declare that the research was conducted in the absence of any commercial or financial relationships that could be construed as a potential conflict of interest.
