# Supplementary material for: Topic Specificity and Antecedents for Preservice Biology Teachers’ Anticipated Enjoyment for Teaching About Socioscientific Issues: Investigating Universal Values and Psychological Distance
Source: Front Psychol. 2020 Jul 24;11:1536. doi: 10.3389/fpsyg.2020.01536 (PMC7393926; doi:10.3389/fpsyg.2020.01536)
Supplement: Supplementary file 1 [file Data_Sheet_1.ZIP › Buessing_et_al_supplementary.docx]

Supplementary Material

# Description of the items

The following table includes all used items for the latent variables of universalism, benevolence, psychological distance, and anticipated enjoyment for teaching in their German and English version.

**Table 1.** Overview of the English and German versions of all items.

| Item | English | German |
| --- | --- | --- |
| **Universalism** | | |
| PVQ01 | ﻿He thinks it is important that every person in the world be treated equally. He believes everyone should have equal opportunities. | Es ist mir wichtig, dass alle Menschen auf der Welt gleichbehandelt werden. Jeder Mensch sollte im Leben die gleichen Chancen haben. |
| PVQ02 | ﻿He believes all the worlds’ people should live in harmony. Promoting peace among all groups in the world is important to him. | Ich glaube, dass die Völker der Welt in Harmonie zusammenleben sollten. Es ist mir wichtig, den Frieden zwischen allen Gruppen der Welt zu fördern. |
| PVQ03 | ﻿It is important to him to adapt to nature and to fit into it. He believes that people should not change nature. | Es ist mir wichtig, mich der Natur anzupassen und zu ihr zu passen. Ich glaube, dass die Menschen die Natur nicht verändern sollten. |
| PVQ04 | ﻿It is important to him to listen to people who are different from him. Even when he disagrees with them, he still wants to understand them. | Es ist mir wichtig, den Menschen zuzuhören, die anders sind als ich. Auch wenn ich anderer Meinung bin als andere, will ich sie trotzdem verstehen. |
| PVQ05 | ﻿He strongly believes that people should care for nature. Look- ing after the environment is important to him. | Ich bin fest davon überzeugt, dass die Menschen sich um die Natur kümmern sollten. Umweltschutz ist mir wichtig. |
| PVQ06 | ﻿He wants everyone to be treated justly, even people he doesnt know. It is important to him to protect the weak in society. | Ich möchte, dass jeder gerecht behandelt wird, sogar Leute, die ich nicht kenne. Es ist mir wichtig, die Schwachen in der Gesellschaft zu beschützen. |
| **Benevolence** | | |
| PVQ07 | ﻿It is important to him to respond to the needs of others. He tries to support those he knows. | Mir ist es wichtig auf die Bedürfnisse der anderen einzugehen. Ich bemühe mich, die Menschen, die ich kenne, zu unterstützen. |
| PVQ08 | ﻿It’s very important to him to help the people around him. He wants to care for their well-being. | Mir ist es wichtig, den Menschen in meinem Umfeld zu helfen. Ich möchte mich um ihr Wohlbefinden kümmern. |
| PVQ09 | ﻿Forgiving people who have hurt him is important to him. He tries to see what is good in them and not to hold a grudge. | Mir ist es wichtig, Menschen zu verzeihen, die mich verletzt haben. Ich versuche, in Ihnen das Gute zu sehen und nicht nachtragend zu sein. |
| PVQ10 | ﻿It is important to him to be loyal to his friends. He wants to devote himself to people close to him. | Mir ist es wichtig, meinen Freunden treu zu sein. Ich möchte mich den Menschen, die mir nahestehen, widmen. |
| **Psychological distance wolf** | | |
| WPN01 | I am personally concerned by the return of wolves. | Ich bin persönlich von der Rückkehr der Wölfe betroffen. |
| WPN02 | ﻿I am concerned by the return of the wolves in my geographical surroundings. | Ich bin in meinem geographischen Umfeld von der Rückkehr der Wölfe betroffen. |
| WPN03 | ﻿I am concerned by the return of the wolves in the near future. | Ich bin in nächster Zeit von der Rückkehr der Wölfe betroffen. |
| WPN04 | ﻿The return of the wolves is very likely | Die Rückkehr der Wölfe ist sehr wahrscheinlich. |
| **Psychological distance climate change** | | |
| CPN01 | I am personally concerned by climate change. | Ich bin persönlich von der Klimaerwärmung betroffen. |
| CPN02 | ﻿I am concerned by climate change in my geographical surroundings. | Ich bin in meinem geographischen Umfeld von der Klimaerwärmung betroffen. |
| CPN03 | ﻿I am concerned by climate change in the near future. | Ich bin in nächster Zeit von der Klimaerwärmung betroffen. |
| CPN04 | ﻿Climate change is likely | Die Klimaerwärmung ist sehr wahrscheinlich. |
| **Psychological distance pre-implantation genetic diagnosis** | | |
| PPN01 | I am personally concerned by pre-implantation genetic diagnosis. | Ich bin persönlich von der Präimplantationsdiagnostik betroffen. |
| PPN02 | ﻿I am concerned by pre-implantation genetic diagnosis in my geographical surroundings. | Ich bin in meinem geografischen Umfeld von der Präimplantationsdiagnostik betroffen. |
| PPN03 | ﻿I am concerned by pre-implantation genetic diagnosis in the near future. | Ich bin in nächster Zeit von der Präimplantationsdiagnostik betroffen. |
| PPN04 | ﻿ It is likely that pre-implantation genetic diagnosis will be allowed in Germany without regulations. | Es ist wahrscheinlich, dass Präimplantationsdiagnostik in Deutschland ohne Einschränkungen erlaubt wird. |
| **Anticipated enjoyment teaching about the topic of returning wolves** | | |
| WEMOJOY1 | I generally enjoy teaching the topic of the return of the wolf. | Im Allgemeinen macht mir das Unterrichten des Themas die Rückkehr des Wolfes Freude. |
| WEMOJOY2 | I generally have so much fun teaching about the topic of the return of the wolf that I gladly prepare and teach my lessons. | Im Allgemeinen macht mir das Unterrichten des Themas die Rückkehr der Wölfe so viel Spaß, dass ich den Unterricht gerne vorbereite und durchführe. |
| WEMOJOY3 | I often have reasons to be happy while I teach about the return of the wolf. | Während des Unterrichtens des Themas die Rückkehr des Wolfes habe ich Grund mich zu freuen. |
| WEMOJOY4 | I generally teach about the return of the wolf with enthusiasm. | Im Allgemeinen unterrichte ich das Thema die Rückkehr des Wolfes mit Begeisterung. |
| **Anticipated enjoyment teaching about the topic of climate change** | | |
| CEMOJOY1 | I generally enjoy teaching the topic of climate change. | Im Allgemeinen macht mir das Unterrichten des Themas Klimaerwärmung Freude. |
| CEMOJOY2 | I generally have so much fun teaching about the topic of climate change that I gladly prepare and teach my lessons. | Im Allgemeinen macht mir das Unterrichten des Themas Klimaerwärmung so viel Spaß, dass ich den Unterricht gerne vorbereite und durchführe. |
| CEMOJOY3 | I often have reasons to be happy while I teach about the topic of climate change. | Während des Unterrichtens des Themas Klimaerwärmung habe ich Grund mich zu freuen. |
| CEMOJOY4 | I generally teach about the topic of climate change with enthusiasm. | Im Allgemeinen unterrichte ich das Thema Klimaerwärmung mit Begeisterung. |
| **Anticipated enjoyment teaching about the topic of pre-implantation genetic diagnosis** | | |
| PEMOJOY1 | I generally enjoy teaching the topic of pre-implantation genetic diagnosis. | Im Allgemeinen macht mir das Unterrichten des Themas Präimplantationsdiagnostik Freude. |
| PEMOJOY2 | I generally have so much fun teaching about the topic of pre-implantation genetic diagnosis that I gladly prepare and teach my lessons. | Im Allgemeinen macht mir das Unterrichten des Themas Präimplantationsdiagnostik so viel Spaß, dass ich den Unterricht gerne vorbereite und durchführe. |
| PEMOJOY3 | I often have reasons to be happy while I teach about the topic of pre-implantation genetic diagnosis. | Während des Unterrichtens des Themas die Präimplantationsdiagnostik habe ich Grund mich zu freuen. |
| PEMOJOY4 | I generally teach about the topic of pre-implantation genetic diagnosis with enthusiasm. | Im Allgemeinen unterrichte ich das Thema Präimplantationsdiagnostik mit Begeisterung. |

# Supplementary factor analyses

Similar to the factorial analysis in the main paper, we also performed confirmatory factor analyses of the scales for the universal values of and psychological distance. The results of these analyses are displayed in Table 2.

**Table 2.** Results from confirmatory factor analysis (CFA) for the respective final models (in bold) and alternative models for psychological distance and universal values with fit indices from Chi-square test (χ^2^) with degrees of freedom (*df*), robust comparative fit index (CFI), robust Root Mean Square Error of Approximation (RMSEA), and Standardized Root Mean Square Residual (SRMR).

|  |  | Fit indices | | | |
| --- | --- | --- | --- | --- | --- |
| Variable | Model | χ^2^ (*df*) | CFI | RMSEA | SRMR |
| Psychological distance (PD) | **Theoretical model: topic-specific**  **(PD_Wolf_ \| PD_CC_ \| PD_PGD_)** | **79.239 (51)** | **.97** | **.06** | **.06** |
|  | Alternative model 1: domain-specific  (PD_Wolf_ + PD_CC_ \| PD_PGD_) | 381.211 (53) | .61 | .20 | .18 |
|  | Alternative model 2: one factor  (PD_Wolf_ + PD_CC_ + PD_PGD_) | 564.169 (54) | .36 | .25 | .21 |
| Universal values of universalism (UNIV) and benevolence (BENE) | Theoretical model  (UNIV \| BENE) | 84.335 (34) | .88 | .09 | .09 |
|  | **Modified model:**  **(UNIV^MOD^ \| BENE)** | **39.810 (26)** | **.96** | **.06** | **.06** |
|  | Alternative model: one factor  (UNIV + BENE) | 151.177 (35) | .72 | .14 | .10 |

Note. _Wolf_ = Topic of returning wolves, _CC_ = Topic of climate change, _PGD_ = Topic of pre-implantation genetic diagnosis, ^MOD^ = factor was modified by excluding one item (PVQ02).
